# Vitamin D Alleviates Rotavirus Infection through a Microrna-155-5p Mediated Regulation of the TBK1/IRF3 Signaling Pathway In Vivo and In Vitro

**DOI:** 10.3390/ijms20143562

**Published:** 2019-07-21

**Authors:** Ye Zhao, Zhiming Ran, Qin Jiang, Ningming Hu, Bing Yu, Li Zhu, Linyuan Shen, Shunhua Zhang, Lei Chen, Hong Chen, Jun Jiang, Daiwen Chen

**Affiliations:** 1 College of Animal Science and Technology, Sichuan Agricultural University, Chengdu 611130, China; 2 Key Laboratory for Animal Disease-Resistance Nutrition of China Ministry of Education, Sichuan Agricultural University, Ya’an 625014, China; 3 Institute of Animal Nutrition, Sichuan Agricultural University, Ya’an 625014, China

**Keywords:** VD, rotavirus, TBK1/IRF3, microRNA-155-5p, pigs, IPEC-J2

## Abstract

(1) Background: Vitamin D (VD) plays a vital role in anti-viral innate immunity. However, the role of VD in anti-rotavirus and its mechanism is still unclear. The present study was performed to investigate whether VD alleviates rotavirus (RV) infection through a microRNA-155-5p (miR-155-5p)-mediated regulation of TANK-binding kinase 1 (TBK1)/interferon regulatory factors 3 (IRF3) signaling pathway in vivo and in vitro. (2) Methods: The efficacy of VD treatment was evaluated in DLY pig and IPEC-J2. Dual-luciferase reporter activity assay was performed to verify the role of miR-155-5p in 1α,25-dihydroxy-VD_3_ (1,25D3) mediating the regulation of the TBK1/IRF3 signaling pathway. (3) Results: A 5000 IU·kg^–1^ dietary VD_3_ supplementation attenuated RV-induced the decrease of the villus height and crypt depth (*p* < 0.05), and up-regulated TBK1, IRF3, and IFN-β mRNA expressions in the jejunum (*p* < 0.05). Incubation with 1,25D3 significantly decreased the RV mRNA expression and the RV antigen concentration, and increased the TBK1 mRNA and protein levels, and the phosphoprotein IRF3 (p-IRF3) level (*p* < 0.05). The expression of miR-155-5p was up-regulated in response to an RV infection in vivo and in vitro (*p* < 0.05). 1,25D3 significantly repressed the up-regulation of miR-155-5p in vivo and in vitro (*p* < 0.05). Overexpression of miR-155-5p remarkably suppressed the mRNA and protein levels of TBK1 and p-IRF3 (*p* < 0.01), while the inhibition of miR-155-5p had an opposite effect. Luciferase activity assays confirmed that miR-155-5p regulated RV replication by directly targeting TBK1, and miR-155-5p suppressed the TBK1 protein level (*p* < 0.01). (4) Conclusions: These results indicate that miR-155-5p is involved in 1,25D3 mediating the regulation of the TBK1/IRF3 signaling pathway by directly targeting TBK1.

## 1. Introduction

Rotavirus (RV), a double-stranded RNA (dsRNA) icosahedral RNA virus, is a major cause of severe diarrhea in infants, young children, and suckling and weaned piglets, which was responsible for nearly 600,000 deaths of children globally every year [1,2,3]. Differentiated epithelial cells on villi in the small intestine are the main targets of infection, leading to cell death, a reduction in the villus epithelium area, loss of absorptive capacity and osmotic dysregulation [4,5,6]. 

After cell entry, retinoic acid-inducible gene I (RIG-I) responds to RV infection by signaling through the mitochondrial antiviral signaling adaptor (IPS-1), which results in an activation of host transcription factors, including interferon regulatory factor 3 (IRF3) and NF-κB, that orchestrate an early IFN-independent transcriptional program [7,8,9]. The TANK-binding kinase 1 (TBK1) is one of the downstream transcription factors of IPS-1. Activated TBK1 then phosphorylates IRF3, triggers their dimerization and nuclear translocation, where they form active transcriptional complexes that bind to IFN stimulation response elements and activate type I IFN (IFN-I) genes expression [10,11].

The VD_3_, a steroid hormone, is hydroxylated in liver and kidney to form 1a,25-dihydroxy VD_3_ (1,25D3), which exhibits several functions, such as the regulation of cell growth and differentiation, embryonic development, metabolic homeostasis and cancer development, through the activation of a VD receptor [12,13]. Newer evidence suggests that it plays a critical role in regulating immune responses to viral infections [14,15,16]. Recently, we have demonstrated that VD could activate the RIG-I and autophagy signaling pathway, thus alleviating the negative effects caused by RV challenge, indicating that VD has an anti-RV infection effect [9,17,18]. Although the role of VD in anti-RV infection is well established, the mechanism of its antiviral action is not fully understood.

MicroRNA (miRNA) are highly conserved, non-coding RNA sequences acting primarily as translational repressors of messenger RNA (mRNA) by interacting with their 3’untranslated region [19]. Recently, several studies have demonstrated that miRNAs always serve as the regulators of an anti-viral immune response, by post-transcriptionally regulating gene and/or protein expression [20,21,22]. Cellular miRNAs play an essential role in the host’s immune responses, especially those of IFN-I, for defending against viral infections [23,24,25,26]. Chanda et al. found that RV up-regulated miR-142-5p expression, which plays the antiviral function during RV infection [27]. Zhang et al. had confirmed that miR-525-3p mediated the antiviral defense to RV infection by targeting nonstructural protein 1 [28]. Thus, miRNA plays vital roles in defense to RV infection. The RV is a dsRNA icosahedral RNA virus. The dsRNA analog poly(I:C) was usually used to probe the mechanisms of the intestinal epithelial cells’ response to dsRNA. The miR-155-5p is one of the best known miRNAs, which was shown to up-regulate after poly(I:C) stimulation or vesicular stomatitis virus challenge [26,29]. Pareek et al. demonstrated that miR-155-5p suppressed Japanese encephalitis virus replication and negatively modulated innate immune response [30]. Moreover, Wang et al. reported that miR-155-5p feedback positively regulated host antiviral innate immune response by promoting IFN-I signaling via the targeting suppressor of cytokine signaling 1 (SOCS1) [31]. However, whether miR-155-5p was involved in RV infection is still unclear. Recently, regulation of miRNA expression by nutrients has been recognized gradually [32,33]. The miRNA expression has been reported to be regulated by 1,25D_3_ [21,33,34]. Nevertheless, studies on the regulation of miR-155-5p by 1,25D3 in anti-RV immune remains unknown.

Thus, we investigate for the first time the effect of 1,25D3 on an RV-induced miR-155-5p expression in vivo and in vitro, and the involvement of miR-155-5p in a 1,25D3-mediated regulation of the TBK1/IRF3 signaling pathway.

## 2. Results

### 2.1. 1,25D3 Inhibits RV Infection via TBK1/IRF3 Signaling Pathway In Vivo and In Vitro

**Figure 1:**
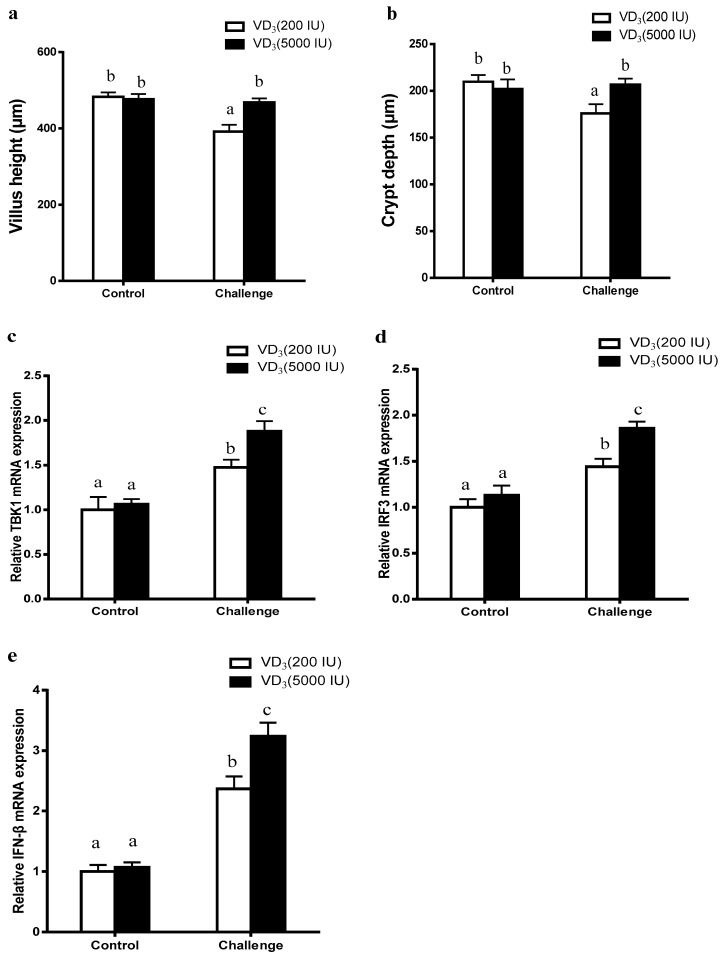
Effect of rotavirus (RV) challenge and vitamin D_3_ (VD_3_) supplement on the villus height (**a**), crypt depth (**b**), and TBK1 (**c**), IRF3 (**d**), and IFN-β (**e**) mRNA expressions in the porcine jejunum. Data are present as the mean ± SD, *n* = 6/group. ^a,b,c^: Means with different letters on vertical bars indicate significant differences (*p* < 0.05).

Data obtained for fecal consistency [17], villus height and crypt depth are shown in Figure 1a,b. The RV challenge increased the fecal consistency (*p* < 0.05) and decreased the villus height and the crypt depth (*p* < 0.05) [17]. Dietary supplementation with 5000 IU VD_3_ decreased the fecal consistency of the RV-challenged pigs, and mitigated the challenge-induced damage to the intestinal epithelium (*p* < 0.05) [17]. The relative expressions of TBK1, IRF3, and IFN-β were presented in Figure 1c–e. The TBK1, IRF3, and IFN-β mRNA expressions were significantly up-regulated by the RV challenge in the porcine jejunum (*p* < 0.05). Dietary supplementation of 5000 IU·kg^−1^ VD_3_ had significantly increased in the TBK1, IRF3, and IFN-β expressions in jejunum (*p* < 0.05). A significant interaction effect of VD_3_ and RV infection was observed on IFN-β mRNA expression.

**Figure 2:**
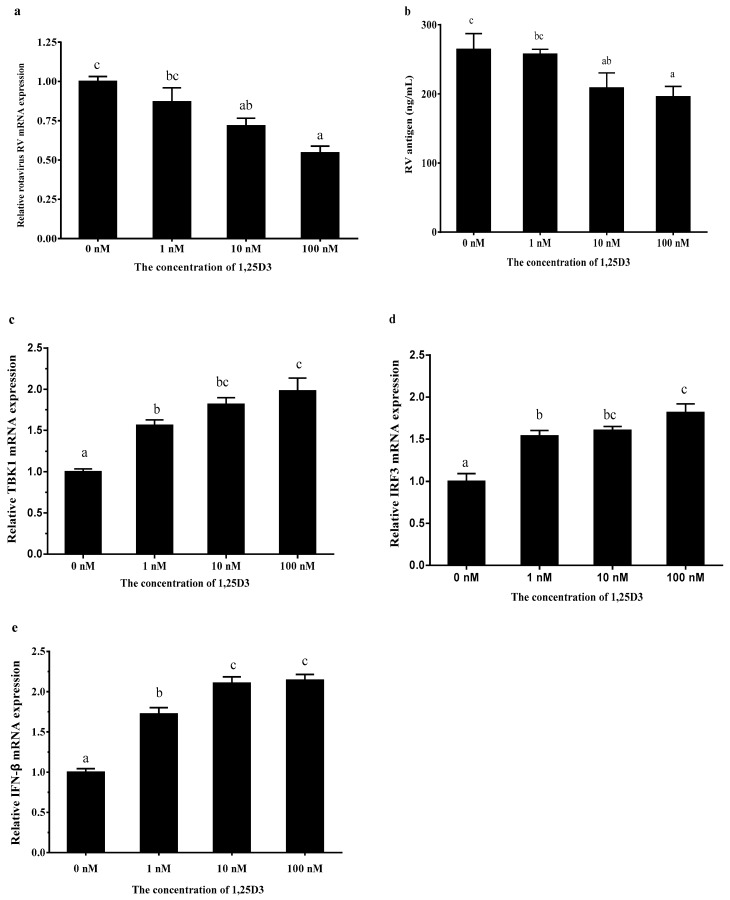

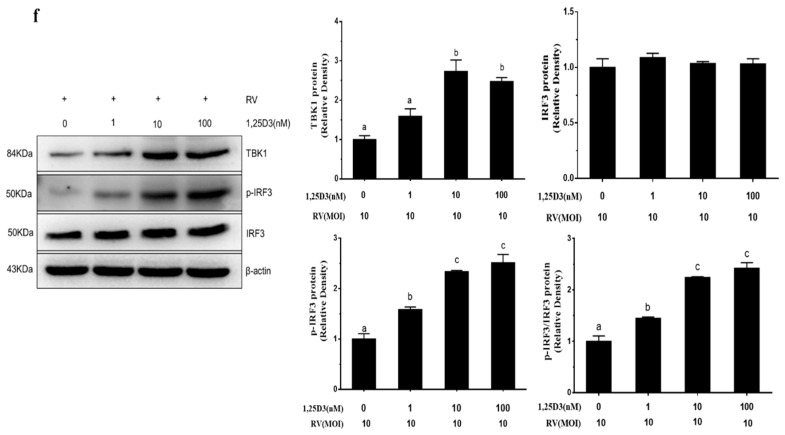
1,25D3 inhibits RV infection via the TBK1/IRF3 signaling pathway in RV-infected IPEC-J2. The effect of 1,25D3 on RV (**a**), RV antigen (**b**), TBK1(**c**), IRF3 (**d**), and IFN-β (**e**) mRNA expressions, and TBK1, IRF3, and p-IRF3 (**f**) protein levels. All data were expressed as means ± SD (bars represent the SD from three independent experiments). ^a,b,c^: Means with different letters on vertical bars indicate significant differences (*p* < 0.05).

To evaluate the effect of 1,25D3 on RV infectivity, RV mRNA expression in cells and RV antigen concentration in the RV-infected cell supernatants were measured. The 1,25D3 (10 and 100 nM) decreased the RV mRNA expression (Figure 2a, *p* < 0.05) and the concentration of RV antigen (Figure 2b, *p* < 0.05). As shown in Figure 2c–e, 1,25D3 (1, 10, and 100 nM) increased the TBK1, IRF3, and IFN-β mRNA expressions in RV-infected IPEC-J2 (*p* < 0.05). Meanwhile, 1,25D3 increased the TBK1 and phosphoprotein IRF3 (p-IRF3) protein levels and p-IRF3/IRF3 (Figure 2f, *p* < 0.05).

### 2.2. 1,25D3 Represses miR-155-5p Expression In Vivo and In Vitro

**Figure 3:**
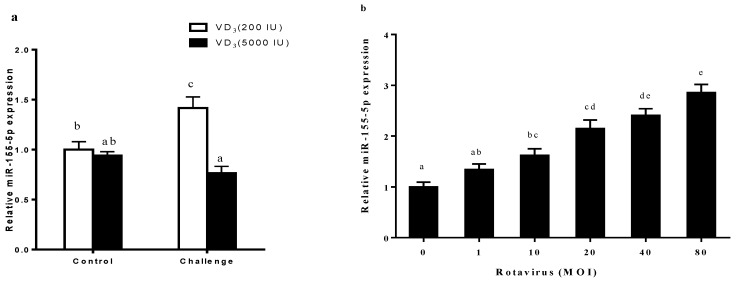

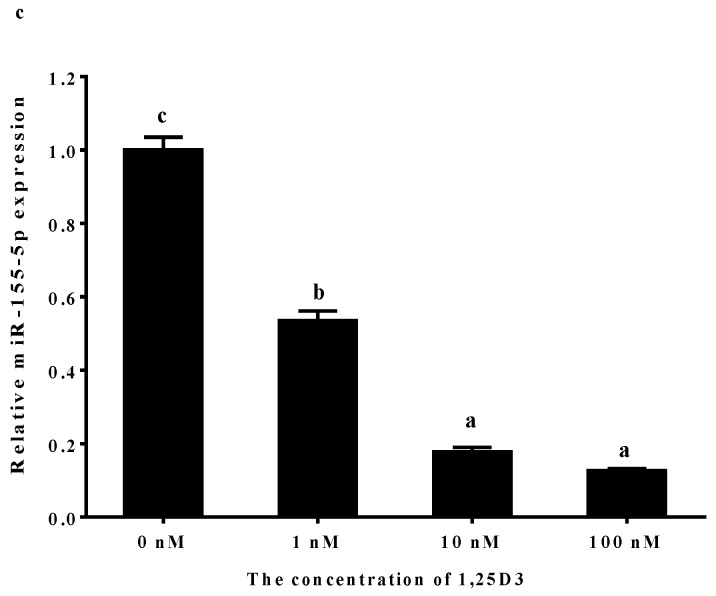
1,25D3 inhibits RV-induced miR-155-5p expression in vivo and in vitro. The effect of RV challenge and VD_3_ supplement on the miR-155-5p expression in the porcine jejunum (**a**), RV MOI on miR-155-5p expression (**b**), 1,25D3 on miR-155-5p expression in RV-infected IPEC-J2 (**c**). All data were expressed as means ± SD (bars represent the SD from three independent experiments). ^a,b,c^: Means with different letters on vertical bars indicate significant differences (*p* < 0.05).

The relative expression of miR-155-5p is shown in Figure 3a. The miR-155-5p expression was significantly up-regulated by the RV challenge in porcine jejunum (*p* < 0.05). Dietary supplementation of 5000 IU·kg^−1^ VD_3_ caused a significant decrease in miR-155-5p expression (*p* < 0.05). A significant interaction effect of VD_3_ and RV infection was observed on miR-155-5p expression (*p* < 0.05).

To explore the expression of miR-155-5p during RV infection, IPEC-J2 cells were infected with RV. The RV-infected cells responded by gradually increasing their expression of miR-155-5p in multiplicity of infection (MOI)-dependent manner at 24 h (Figure 3b, *p* < 0.05). 1,25D3 significantly repressed the up-regulation of miR-155-5p (Figure 3c, *p* < 0.05). These results clearly indicated that VD repressed any miR-155-5p up-regulation that was induced by RV infection in vivo and in vitro. This apparent association of miR-155 up-regulation with RV infection and down-regulation by 1,25D3 prompted us to investigate further the role of miR-155-5p in RV infection.

### 2.3. miR-155-5p Is Involved in 1,25D3 Inhibited RV Infection

**Figure 4:**
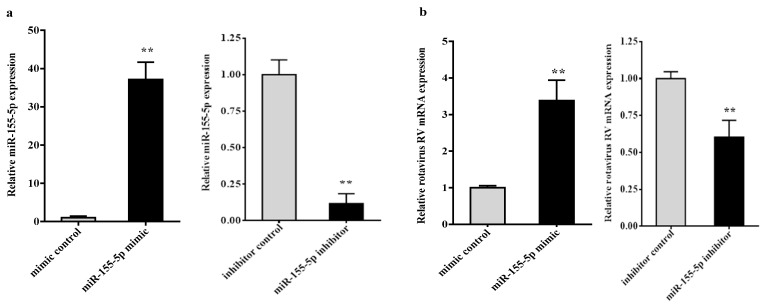

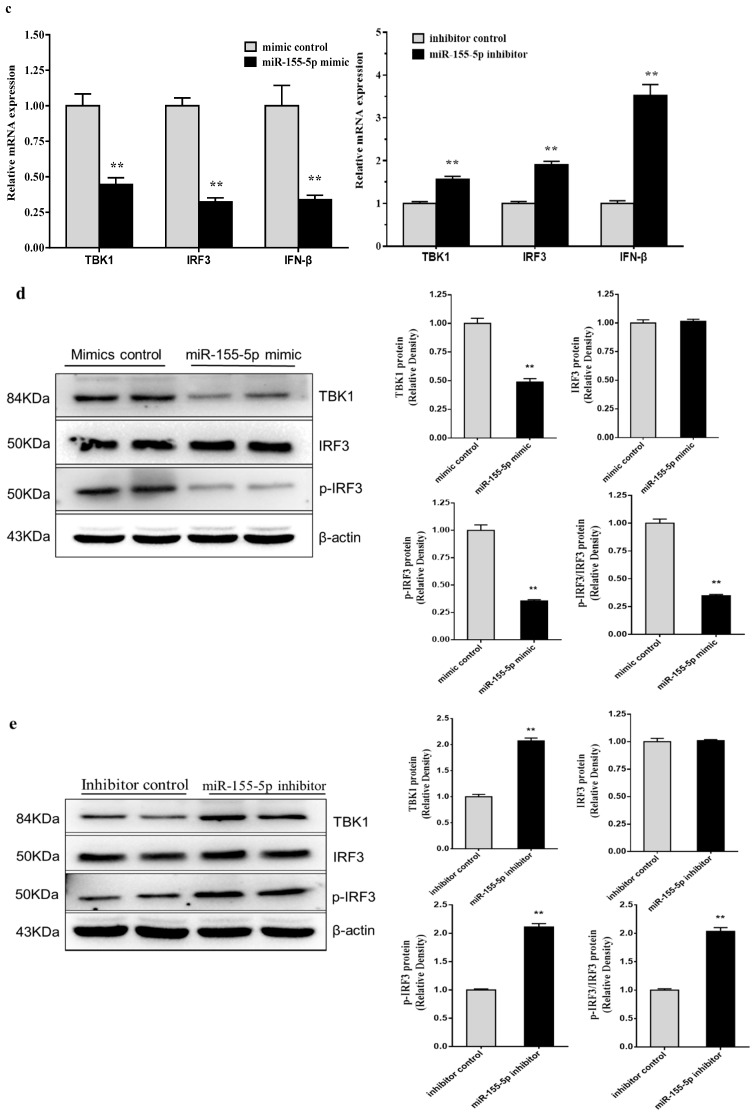
This miR-155-5p is involved in RV infection process. Cells were transfected with miR-155-5p mimic or inhibitor, and then infected with RV at a MOI of 10 for 1 h. The miR-155-5p expression (**a**), RV (**b**), TBK1, IRF3, and IFN-β mRNA levels (**c**) were measured by real-time quantitative PCR. The TBK1, IRF3 and p-IRF3 protein levels in the IPEC-J2 cells treated with miR-155-5p mimic (**d**) or inhibitor (**e**) were measured by western blot. All data were expressed as means ± SD (bars represent the SD from three independent experiments). ** *p* < 0.01.

To determine whether miR-155-5p plays a role in RV infection, we transfected miR-155-5p mimics, miRNA mimics negative control, miR-155-5p inhibitor or miRNA inhibitor negative control into IPEC-J2, then cells were infected with RV at 10 MOI. As shown in Figure 4a, the level of miR-155-5p was increased in cells transfected with miR-155-5p mimic (*p* < 0.01), compared to miRNA mimic negative control, whereas miR-155-5p inhibitor decreased the level of miR-155-5p (*p* < 0.01), indicating that the transfection was efficient. Under these conditions, an overexpression of miR-155-5p increased the RV mRNA expression (Figure 4b, *p* < 0.01). In contrast, the inhibition of miRNA-155-5p decreased our RV mRNA level (Figure 4b, *p* < 0.01). Furthermore, to confirm the effect of miR-155-5p on the TBK1/IRF3 signaling pathway, we also detected the expression levels of TBK1, IRF3 and IFN-β. As shown in Figure 4c, overexpression of miR-155-5p remarkably suppressed the mRNA expression levels of TBK1, IRF3, and IFN-β (*p* < 0.01), while the inhibition of miR-155-5p enhanced the expressions of these genes (*p* < 0.01) compared to our control group. Western blot analysis showed that the TBK1 protein level and p-IRF3/IRF3 were decreased after miR-155-5p mimic treatment, while their expression levels were increased with miR-155-5p inhibitor treatment, compared to that in the control group (Figure 4d,e, *p* < 0.01).

### 2.4. 1,25D3 Inhibits RV Infection via miR-155-5p by Targeting TBK1

**Figure 5:**
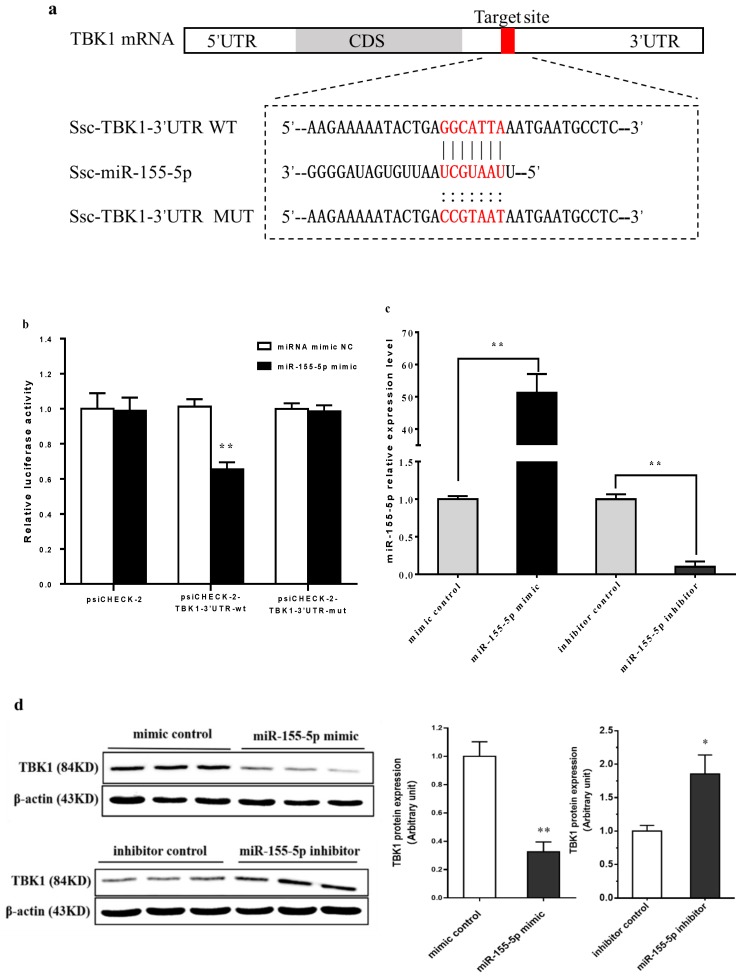
The miR-155-5p targets the 3’-UTR of TBK1. Sequence alignment of wild-type (WT) and mutated (MUT) putative miR-155-5p-binding sites in the 3’-UTR of TBK1 (**a**). Binding site and seed region of miR-155-5p are indicated in red. The repressive effect of miR-155-5p on the activity of TBK1 3’UTR was measured by dual-luciferase reporter activity (**b**). Sequence of WT binding site is GGCATTA, and the binding site of MUT is CCGTAAT. The influence of miR-155-5p mimic and inhibitor on miR-155-5p expression (**c**). The influence of miR-155-5p mimic and inhibitor on TBK1 protein expression (**d**). All data were expressed as means ± SD (bars represent the SD from three independent experiments). * *p* < 0.05, ** *p* < 0.01.

To further explore the potential mechanism underlying whether the miR-155-5p was involved in a 1,25D3-mediatied inhibition of RV infection, we used computational prediction programs, like targetScan, pictar, and miRanda, to predict the potential target genes of miR-155-5p. The potential target genes of miR-155-5p were shown in Table 1. The TBK1, which plays an important role in RV infection by directly inducing IFN-β expression, is one of the candidate target genes. As shown in Figure 5a, TBK1 was identified as a potential target gene of miR-155-5p, with the predicted binding site at the base from positions 50 to 56. This possibly implies the epigenetic regulation. Furthermore, we constructed wild type (WT) and mutant (MUT) double fluorescent reporter gene plasmids to confirm whether miR-155-5p suppressed TBK1 activity by binding to its 3’-UTR. Decrease of the relative luciferase activity was observed in Hela cells co-transfected with WT luciferase plasmid and miR-155 mimic (Figure 5b, *p* < 0.01). However, no reduction in luciferase activity was observed in cells cotransfected with the MUT luciferase plasmid (Figure 5b, *p* > 0.05). As shown in Figure 5c, the level of miR-155-5p was significantly increased in cells transfected with miR-155-5p mimic (*p* < 0.01), compared to miRNA mimic negative control, whereas the miR-155-5p inhibitor decreased the level of miR-155-5p (*p* < 0.01), indicating that the transfection was efficient. Moreover, we also found that miR-155-5p suppressed the TBK1 protein level (*p* < 0.01) (Figure 4d). Taken together, these data suggest that miR-155-5p inhibits RV infection by directly targeting the 3’-UTR of TBK1.

## 3. Discussion

The IPEC-J2 cell line, derived from the jejunum epithelium of a neonatal un-suckled piglet, is a non-transformed and non-tumorigenic porcine small intestine cell line [35,36]. Therefore, it is an appropriate model for researching the innate immune responses to RV [37]. The IFN-I mediates antiviral activity by triggering the expression of IFN-stimulated genes, which products have diverse antiviral activities [38,39]. The RIG-I/IPS-1 signaling is the main arm of the innate immunity upon RV infection, and has a functionally important role in determining the magnitude of RV replication in the intestinal epithelium [7,40]. The TBK1, a downstream transcription factor of IPS-1, mediates the activation of IRF3, leading to the induction of IFN-I following viral infections [11,41,42], and is also tightly regulated to effectively control viral infections and maintain immune homeostasis [43]. The present result showed that the RV challenge caused a reduction in villus height and crypt depth. The RV replicates in the enterocytes of the small intestine, inducing villus atrophy [44,45]. In addition, the mRNA expressions of TBK1 and IRF3 were up-regulated to various degrees after the RV challenge. This is in close agreement with previous reports in mice that TBK1 is required for the activation of innate immune responses to RNA viruses [41,42]. The VD, a well-known steroid hormone that regulates innate immune response, plays a vital role in anti-viral infection [15,16,46]. We have shown that VD decreased the fecal consistency scores of RV-challenged pigs, which have powerful anti-RV effects in vivo and in vitro [9,17,18]. Specifically, we found that RIG-I signaling, as well as autophagy signaling, are required for this inhibitory effect [9,17]. Recent studies demonstrated that VD might enhance antiviral defense to rhinovirus and dengue virus infection via innate interferon pathways [14,16,47]. These studies also revealed that the antiviral impact of VD in vivo and in vitro models was largely due to the production of IFN-β [9,17]. In the current work, we sought to explain the mechanism that leads to the large burst of IFN that protects the host from infection. The present results showed dietary supplementation of 5000 IU VD_3_ enhanced RV-induced up-regulation of TBK1, IRF3 and IFN-β mRNA expressions. 1,25D3 increased the TBK1 and p-IRF3 protein levels, and p-IRF3/IRF3. These results indicated that 1,25D3 alleviated RV infection through the regulation of the TBK1/IRF3 signaling pathway.

The miRNA are a class of endogenous non-coding RNA that play critical roles in the regulation of immune response [48,49]. A growing body of evidence supports the idea that abnormal expression of miRNAs is required for the initiation and procession of viral infection through the regulation of innate immune response [50,51,52]. The current study firstly identified that the expression of miR-155-5p was up-regulated in response to RV infection in vivo and in vitro. The VD significantly suppressed the up-regulation of miR-155-5p. Correlation analysis showed that the VD was negatively related to miR-155-5p, suggesting that miR-155-5p may be involved in the anti-viral effect of VD. To elucidate the potential effect of miR-155-5p on RV infection, IPEC-J2 were transiently transfected with miR-155-5p mimic or inhibitor, respectively. The present results showed the transfection of miR-155-5p mimic in IPEC-J2 increased RV mRNA expression, whereas the transfection of the miR-155-5p inhibitor gave an opposite result, indicating that miR-155-5p is involved in RV infection. As a key kinase in antiviral immune responses, TBK1 directly mediates the phosphorylation of IRF3, then regulates the production of IFN-β production [53,54]. Gene deletion studies demonstrate that the TBK1 (to a lesser extent) functions redundantly in its phosphorylation of IRF3 in various types of cells [54,55]. To further confirm the effect of miR-155-5p on RV infection, we also detected the expression levels of RV, TBK1, IRF and IFN-β. Overexpression of miR-155-5p remarkably suppressed the mRNA expressions of RV, TBK1, IRF3 and IFN-β, while the inhibition of miR-155-5p enhanced the expressions of these genes. Western blot analysis showed that the TBK1 protein level and the p-IRF3/IRF3 were significantly decreased after miR-155-5p mimic treatment, while their expression levels were significantly increased with miR-155-5p inhibitor treatment compared to that in our control group. Therefore, these results collectively suggest that miR-155-5p is involved in VD-mediated regulation of the TBK1/IRF3 signaling pathway.

To further explore the potential mechanism underlying the viral infection-inhibiting effect of miR-155-5p, we used computational prediction programs, like targetScan, pictar, and miRanda, to predict the potential target genes of miR-155-5p. The prediction results revealed that most of the predicted target genes were involved in the viral infection process. For instance, the SOCS1 has been identified to negatively regulate various immune responses and signaling pathways, including IFN-I signaling [56]. The Src homology 2-containing inositol phosphatase 1 (SHIP1) has been shown to be a negative regulator of IFN-β production and lipopolysaccharide-induced antibacterial response in mice [57,58]. Myeloid differentiation protein 88 (MyD88) is critical for the development of innate and adaptive immunity during virus infection [59,60]. Thus, the current study primarily focuses on the roles of miR-155-5p in immunity and potential signaling mechanisms. The TBK1, which is one of the candidate target genes of miR-155-5p, is a key adaptor protein for the antiviral immunity signaling pathway. The TBK1 is constitutively expressed, and IRF3 activation is attenuated in TBK1-deficient cells [55]. Previous study showed that miR-221-3p targets kinase TBK1, which is important for interferon production and virus clearance. Down-regulation of TBK1 in the liver will promote viral persistence [11,61]. The present study verified TBK1 as a direct target of miR-155-5p using dual-luciferase reporter gene assay. Overexpression of miR-155-5p could repress the protein level of TBK1, while the inhibition of miR-155-5p enhanced the expression of TBK1. These data suggest that TBK1 is a direct and functional target of miR-155-5p. Several miRNA have been reported to control RV replication [27,62]. This study has added miR-155-5p, which is a new member involved in RV infection.

## 4. Materials and Methods 

### 4.1. Virus

The rotavirus (RV) (purchased from China Institute of Veterinary Drug Control) was a tissue culture-adapted Ohio State University (OSU) strain (ATCC #VR-893), which was passaged in MA104 clone 1 cells (ATCC# CRL-2378.1^TM^). The virus was adapted to growth in IPEC-J2 cells. The virus titer was determined using a 50% tissue culture infective dose (TCID_50_) assay according to our previous study [17], and virus stock was stored at −80 °C.

### 4.2. Animal and Experimental Design

All animal experimental procedures and sample collections were performed in accordance with the guidelines of Institutional Animal Care and Use Committee of the College of Animal Science and Technology of Sichuan Agricultural University, Sichuan, China, under permit NO. DKY-B20131403 (Ministry of Science and Technology, China, revised in June 2004). Twenty four Duroc × (Landrace × Yorkshire) pigs with an initial body weight of 21.82 ± 2.06 kg were used in a 2 × 2 design, the main factors consisted of RV challenged (RV vs. DMEM/F12 medium) and dietary levels of Vitamin D_3_ (VD_3_) (200 vs. 5000 IU·kg^−1^). The pigs were placed individually in metabolic cages (1.5 m × 0.7 m × 1.0 m), provided ad libitum access to feed and water, which were located in a room with a controlled temperature (25 °C ± 1 °C). All diets and feeding management were the same as described in our previous study [17]. Pigs were fed the experimental diets for 7 days before RV challenge. On day 8 of the dietary treatments, all pigs were orally administrated either with the 4 mL DMEM/F12 medium containing RV (10^5.8^TCID_50_/100 μL) or the same amount of the DMEM/F12 medium, and fed their respective diets for 7 days. The pigs were checked daily to evaluate their status after this RV challenge. Clinical signs (i.e., dehydration, apathy and diarrhea) were monitored daily. Fecal consistency was recorded each morning at 08:00 by a visual appraisal of each sample using a four-point scoring system and scored as follows: 0, No diarrhea; 1, stiff flowing feces; 2, easy flowing feces; 3, severe, watery diarrhea. At the end of the experiment, all pigs were sacrificed, the jejunum samples were quickly taken and frozen in liquid nitrogen, then stored at −80 °C for further analysis. Meanwhile, the jejunum was also isolated for histological analysis.

### 4.3. Cell and Treatment

#### 4.3.1. Chemicals and Intestinal Epithelial Cell Line

1,25D3 were purchased from Sigma (St. Louis, MO, USA). Mimic and inhibitor oligonucleotides of miR-155-5p were synthesized by Ribobio (Guangzhou, China). The IPEC-J2 cell line, a kind gift from Per Torp Sangild (University of Copenhagen, Denmark), was cultured as previously described [9].

#### 4.3.2. 1,25D3 Treatments

1,25D3 stock solutions of 10^−2^ M were dissolved in less than 0.1% ethanol and further dilutions were performed using DMEM/F12 medium. Experimental procedures were similar to those previously described in another study conducted in our laboratory [9,18]. Briefly, the cells were inoculated with various concentrations of 1,25D3 (0, 1, 10, and 100 nM) for 24 h at 37 °C with 5% CO_2_, followed by the removal of the medium and the washing of the cells three times with PBS, then challenged with RV at a different MOI for 1 h. Following removal of the inoculums and two washings, the cells were incubated with basal medium (serum free) containing 1,25D3 (0, 1, 10, and 100 mM) for a further 24 h. Then, the cells together with supernatants were frozen and thawed once, and clarified by centrifugation. The supernatants were collected to determine the concentration of RV antigen. Repeating the above procedure, cells were harvested to detect the genes and proteins expression.

#### 4.3.3. Transfection of miRNA Mimic and Inhibitor

When IPEC-J2 reached about 80% confluence, these cells were transiently transfected with 100 nM miR-155-5p mimics, 100 nM mimics negative control, 200 nM miR-155-5p inhibitor or 200 nM miRNA inhibitor negative control. The process of transfection was conducted using the Lipofectamine 3000 (Invitrogen, Carlsbad, CA, USA) according to the manufacturer’s instructions.

### 4.4. Determination of RV Antigen in the Cell Supernatants by ELISA

Concentration of RV antigen in the treated (frozen, thawed and centrifuged) supernatants were measured using the ELISA kits (R&D Systems China Company Limited, Shanghai, China) according to our previous study [18].

### 4.5. Real-Time Quantitative PCR

Total RNA was isolated using TRIZOL reagent (Invitrogen) according to the manufacturer’s instructions. The concentrations of total RNA were determined spectrophotometrically using a Beckman Coulter DU 800 (Beckman Coulter, Fullerton, CA, USA). The integrity of the RNA was verified by agarose gel electrophoresis. Then, mRNA and miRNA were reverse-transcribed to cDNA using a commercial kit (TaKaRa, Dalian, China). Real-time quantitative PCR was performed using a SYBR Premix EX Taq kit (TaKaRa) and the CFX96 Real-Time PCR Detection System (Bio-Rad, Hercules, CA, USA). The relative expression of miRNA and mRNA were calculated using the 2^−ΔΔ*C*t^ method. The β-actin and U6 were used as internal normalizing controls for mRNA and miRNA, respectively. All PCR primer sequences were shown in Table 2.

### 4.6. Luciferase Reporter Assay

The wild-type 3’UTR of TBK1 was amplified from the liver of a pig. The mutant-type TBK1 3’-UTR was obtained using commercial kits (TransGen Biotech, Beijing, China), according to the manufacturer’s instructions. The wild-type and mutant TBK1 3’-UTR were inserted into the psiCHECK™-2 vector (Promega, Madison, WI, USA) between the XhoI and NotI restriction sites respectively. For the luciferase reporter analysis, HeLa cells were respectively transfected with recombinant psiCHECK™-2 vector containing wild-type or mutant TBK1 3’-UTR, and miR-155-5p mimic. After transfection, luciferase activities were measured using the Dual-GloLuciferase Assay System (Promega), following the manufacturer’s instructions.

### 4.7. Western Blot

Protein was extracted from IPEC-J2 cells using lysis buffer (Sigma), according to the manufacturer’s instructions. Samples containing equal amounts of protein were run on 10% SDS-polyacrylamide gel and then transferred to a polyvinyldifluoride membrane (Bio-Rad). After blocking with TBST containing 5% nonfat dried milk for 2 h at 37 °C, the membrane was hybridized with the primary antibody of Cell Signaling (TBK1, catalogue no. 3504; IRF3, catalogue no. 4302; p-IRF3, catalogue no. 4947), incubated overnight at 4 °C, and then washed three times with TBST for 10 min each. After washing, the membrane was incubated with secondary antibody for 1 h at 37 °C, then washed three times with TBS/T for 10 min. Clarity western enhanced chemiluminescence (ECL) substrate (Bio-Rad) was used to visualize the signals. The Gel-Pro Analyzer (Media Cybernetics, Inc. Bethesda, MD, USA) was used to quantify protein expression, and the ratio of target proteins’ expression was normalized to β-actin.

### 4.8. Statistical Analysis

Data were analyzed with SPSS (21.0 version). All data were presented as means ± standard deviation (SD). Differences in groups were analyzed with Student’s t-test when there were less than three experiment groups. One-way analysis of variance (ANOVA) was used when there were more than three experiment groups. *p* < 0.05 was considered to be statistically significant.

## 5. Conclusions

This study provided the first evidence that VD alleviated RV infection through the TBK1/IRF3 signaling pathway and miR-155-5p in porcine intestine and IPEC-J2 cells. In addition, miR-155-5p is involved in the 1,25D3-mediated regulation of the TBK1/IRF3 signaling pathway by directly targeting TBK1. These findings suggested a net beneficial effect of 1,25D3 in RV infection.

## Figures and Tables

**Table 1:** The potential target genes of miR-155-5p.

Target Gene	Gene Description
TBK1	TANK-binding kinase 1
SOCS1	Suppressor of cytokine signaling 1
SHIP1	Src homology 2-containing inositol phosphatase 1
TAB2	TGF-β activated kinase 1/MAP3K7 binding protein 2
MyD88	Myeloid differentiation protein 88
MITF	Microphthalmia-associated transcription factor
RICTOR	Rapamycin-insensitive companion of mTOR
TBRG1	Transforming growth factor beta regulator 1
JARID2	Jumonji, AT rich interactive domain 2
C/EBPβ	CCAAT/enhancer-binding protein beta
PU.1	Transcription factor that binds to the PU-box, a purine-rich DNA sequence
FOS	FBJ murine osteosarcoma viral oncogene homolog
IRF2BP2	Interferon regulatory factor 2 binding protein 2
SMAD2	SMAD family member 2
SMAD5	SMAD family member 5
CTLA-4	Cytotoxic T-lymphocyte-associated protein

**Table 2:** Primer sequences and optimal annealing temperatures (OAT, °C) of genes selected for analysis by real-time PCR.

Name	Sequence (5′–3′)	OAT
RV-QF	TCAGTTCGTCAGGAATATGC	53.5
RV-QR	CTTGAAGGTGAGTAGTTGGT	
TBK1-QF	CAGCGTGGCTAAGGCAATAA	63.0
TBK1-QR	CATCGTATCCCCTTTCGCAT	
IRF3-QF	TCATCGAAGATCTGATTGCCTTC	57.2
IRF3-QR	GGGACAACCTTGACCATCACC	
IFN-β-QF	AATCGCTCTCCTGATGTGTT	59.6
IFN-β-QR	TTGCTGCTCCTTTGTTGGTA	
miR-155-5p-QF	CGCGTGTTAATGCTAATTGTGA	55.7
miR-155-5p-QR	AGTGCAGGGTCCGAGGTAT	
β-actin-QF	TCTGGCACCACACCTTCT	59.0
β-actin-QR	TGATCTGGGTCATCTTCTCAC	
U6-QF	CTCGCTTCGGCAGCACA	60
U6-QR	AACGCTTCACGAATTTGCGT	

## Abbreviations

RV	Rotavirus
miR-155-5p	MicroRNA-155-5p
miRNA	MicroRNA
TBK1	TANK-binding kinase 1
IRF3	Interferon regulatory factors 3
1,25D3	1α,25-dihydroxy-vitamin D_3_
RIG-I	Retinoic acid-inducible gene I
IPS-1	Mitochondrial antiviral signaling adaptor
IFN-I	Type I IFN
p-IRF3	Phosphoprotein IRF3
SOCS1	Suppressor of cytokine signaling 1
SHIP1	Src homology 2-containing inositol phosphatase 1
TAB2	TGF-beta activated kinase 1/MAP3K7 binding protein 2
MyD88	Myeloid differentiation protein 88
MITF	Microphthalmia-associated transcription factor
RICTOR	Rapamycin-insensitive companion of mTOR
TBRG1	Transforming growth factor beta regulator 1
JARID2	Jumonji, AT rich interactive domain 2
C/EBPβ	CCAAT/enhancer-binding protein beta
PU.1	Transcription factor that binds to the PU-box, a purine-rich DNA sequence
FOS	FBJ murine osteosarcoma viral oncogene homolog
IRF2BP2	Interferon regulatory factor 2 binding protein 2
SMAD2	SMAD family member 2
SMAD5	SMAD family member 5
CTLA-4	Cytotoxic T-lymphocyte-associated protein
OAT	Optimal annealing temperatures
